# Risk Factors for Mortality in Patients with Aortoesophageal Fistula Related to Aortic Lesions

**DOI:** 10.1155/2020/4850287

**Published:** 2020-09-17

**Authors:** Shan Li, Feng Gao, Hai-ou Hu, Jin Shi, Jie Zhang

**Affiliations:** ^1^Department of Gastroenterology, Beijing Anzhen Hospital, Capital Medical University, Beijing, China; ^2^Beijing Anzhen Hospital, Capital Medical University, No. 5A, Cardiosurgical Ward, Beijing, China

## Abstract

**Objective:**

Aortoesophageal fistula (AEF) related to aortic aneurysm and dissection is an uncommon but life-threatening condition. We performed a systematic review of risk factors for mortality and factors associated with the prognosis of AEF.

**Methods:**

A systematic search of the PubMed, Embase, and Cochrane Library databases was performed. Clinical characteristics, diagnostic methods, and treatments were assessed in terms of their ability to predict mortality.

**Results:**

The systematic review identified 184 eligible articles including 219 patients with AEF. Multivariable Cox regression revealed positive correlations of hemorrhagic shock (hazard ratio (HR): 1.824, 95% CI: 1.217-2.735, *P* = 0.004), sepsis (HR: 1.714, 95% CI: 1.112-2.641, *P* = 0.015), multiorgan failure (HR: 3.060, 95% CI: 1.470-6.368, *P* = 0.003), and conservative treatment (HR: 5.257, 95% CI: 3.405-8.116, *P* < 0.001) with mortality and a negative correlation between combination therapy (aortic graft replacement and esophagectomy) and mortality (HR: 0.319, 95% CI: 0.125-0.813, *P* = 0.017). Kaplan–Meier survival analysis showed that the 1-year cumulative survival rate was 42.5 ± 3.8%. The overall fistula-related mortality rate was 47.0% (103/219). The most common causes of death were bleeding (54.9%) and infection (29.2%).

**Conclusions:**

We found that hemorrhagic shock, sepsis, and multiorgan failure were risk factors for death in patients with AEF. Additionally, conservative treatment was associated with a higher rate of mortality, while combined aortic graft replacement and esophagectomy improved the prognosis.

## 1. Introduction

Aortoesophageal fistula (AEF) usually results in fatal gastrointestinal bleeding and is associated with a high mortality rate, despite the use of advanced surgical and endovascular techniques [1]. Previous studies reported that nearly 60% of patients with AEF died within 6 months; the 1-year survival rate was only 28% [2, 3]. Since the 1990s, thoracic endovascular aortic repair (TEVAR) has been increasingly used as a less invasive treatment for aortic aneurysm and aortic dissection [4]; however, post-TEVAR AEF has been frequently observed as a fatal complication [5]. Due to the rarity of AEF, few studies have investigated its risk factors for mortality, or the long-term prognosis [2]. Therefore, we systematically reviewed the literature on AEF related to aortic lesions, including aortic aneurysm and dissection. We reviewed clinical characteristics, diagnostic methods, treatment options, and prognosis of AEF, especially regarding factors associated with poor outcomes of AEF.

## 2. Methods

### 2.1. Search Strategy

The review was performed following the Preferred Reporting Items for Systematic Reviews and Meta-Analysis (PRISMA) guidelines. A systematic search was undertaken in the PubMed, Embase, and Cochrane Library databases from 2000 to March 6, 2020. The search terms were “aortic aneurysm,” “aneurysm, dissecting,” “endovascular procedures,” “aortic surgery,” “endograft,” “stent graft,” and “esophageal fistula.” Only articles written in English were included. The references of retrieved articles were reviewed manually to identify missed studies.

### 2.2. Eligibility Criteria

Studies were included in accordance with the following eligibility criteria: (1) observational studies, case reports, or case series; (2) sufficient information for analysis including outcomes and management strategies; (3) AEF associated only with aortic aneurysm or dissection; and (4) full-text article or meeting abstract. Duplicate studies were excluded; only the latest version was included in the analysis when the information was inconsistent.

### 2.3. Data Extraction

All articles were independently reviewed, and data were extracted by two reviewers (SL and FG); the final decision regarding inclusion was made by another reviewer (JZ). The following data were analyzed: age and sex; location and size of aortic aneurysm or dissection; a history of aortic surgery and/or endovascular treatment and the interval between the latest procedure and the occurrence of AEF; comorbidity and clinical symptoms (chest/abdominal/back pain, dysphagia, fever, and hemorrhage); elevated inflammatory markers and positive bacterial culture; diagnostic methods and related manifestations; location and size of fistula; treatments; and follow-up duration and prognosis.

### 2.4. Statistical Analysis

Categorical variables are described as percentages and continuous variables as means ± standard deviations or medians. Comparisons of the primary and secondary AEF groups were performed by the Wilcoxon two-sample test, Fisher's exact test, or *χ*^2^ test, as appropriate. The survival rate was evaluated by Kaplan–Meier analysis. Univariable and multivariable Cox regression analyses were conducted to identify factors independently related to the outcomes of interest. We excluded variables from the univariable analysis for multivariable regression model if their differences were not significant between groups and if the percentage of missing data was more than 15% of all the AEF patients. A *P* value of < 0.05 was considered indicative of statistical significance. Statistical analysis was performed using SPSS ver. 21.0 software (IBM®, Armonk, NY).

## 3. Results

**Figure 1:**
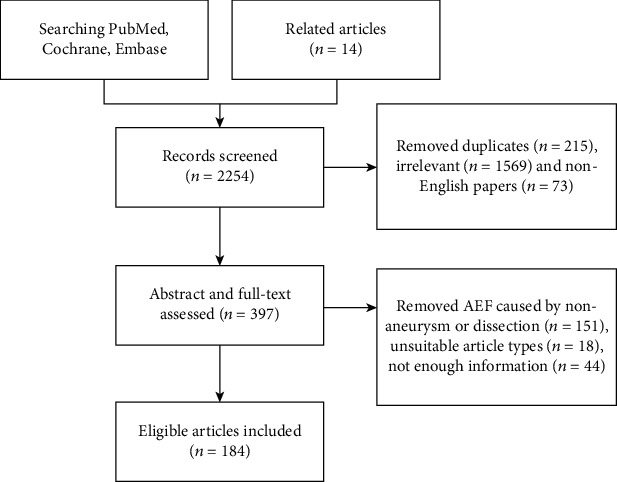
PRISMA flowchart.

The systematic review identified 184 eligible articles that included 219 patients with AEF. The mean patient age was 65.8 ± 13.5 years, and 63.1% of patients were men. Detailed information on the included articles is provided in the supplementary file (available here). The PRISMA flowchart is shown in Figure 1.

### 3.1. Demographic and Clinical Features

The baseline characteristics of the survivors were similar to those of the nonsurvivors, except for age and hemorrhage shock (Table 1). Compared with the nonsurvivors, the survivors were significantly younger (62.4 ± 13.2 vs. 68.9 ± 13.0 years, *P* < 0.001) and less presented frequent hemorrhage shock (37.1% vs. 62.2%, *P* < 0.001). There were no differences in fistula location, radiographic findings, or endoscopic findings between the two groups.

In our study, 7.8% (17/219) of total AEF patients were diagnosed as mycotic aortic aneurysms (AA). 47.1% (8/17) of which were primary AEFs, and 52.9% (9/17) were secondary AEFs which means AEF were confirmed after mycotic AA treated by graft replacement or TEVAR. For mycotic AA, the proportion of primary AEFs was significantly higher in survivors than in nonsurvivors while secondary AEFs took a larger proportion in nonsurvivors (85.7% vs.20.0%, 14.3% vs.80.0%, *P* = 0.015, Table 2). The mortality rate of patients with AEF after primary mycotic AA treated with TEVAR was extremely high (100%, 5/5), but the difference was not statistically significant due to the small sample size (*P* = 0.444). Positive cultures were mentioned in 61 cases, and the top three prevalent agents were *Staphylococcus aureus* (36.1%, 22/61), *Streptococcus* species (21.3%, 13/61), and *Candida* species (21.3%, 13/61).

### 3.2. Treatment

The treatments for aortic and esophageal lesions are shown in Table 3. For aortic lesions, 32.9% of the patients underwent graft replacement, 21.5% underwent TEVAR, and 8.7% underwent both graft replacement and TEVAR. Only the graft replacement rate differed between survivors and nonsurvivors (49.5% vs. 18.1%, *P* < 0.001). Both Dacron graft and cryopreserved aortic allograft were common choices and differed between survivors and nonsurvivors (43.0% vs.14.0%, *P* < 0.001; 15.0% vs. 6.1%, *P* = 0.042, Table 4). Among the 76 patients who received no treatment for aortic lesions, 10 survived, and 66 died (9.7% vs. 56.9%, *P* < 0.001). Regarding esophageal lesions, 27.9% of the patients underwent esophagectomy, 16.0% underwent fistula repair, 10.0% received esophageal stents, and 5.0% received combination esophageal treatments. Only esophagectomy was performed more frequently in survivors than in nonsurvivors (45.6% vs. 12.1%, *P* < 0.001). Among the 90 patients who received no esophageal treatments, 18 survived, and 72 died (17.5% vs. 62.1%, *P* < 0.001). The therapies used in patients with AEF are summarized in Table 2. The combination of aortic graft replacement and esophagectomy was the only therapy performed more frequently in survivors than in nonsurvivors (30.1% vs. 6.0%, *P* < 0.001). In total, 55 of 57 patients who received no treatment for aortic or esophageal lesions had a fatal outcome (mortality rate of 94.5%). Furthermore, 47.8% of patients with a follow-up period longer than 4 weeks received prolonged antibiotic treatment (>4 weeks); however, the difference was not statistically significant between the groups (53.3% vs. 37.0%, *P* = 0.102).

### 3.3. Short-Term Outcomes

The overall in-hospital mortality rate was 42.0% (94/219). Complications were observed in 84 patients (38.4%, Table 1); this rate was significantly lower in survivors than in nonsurvivors (28.2% vs. 47.4%, *P* = 0.004). Sepsis (23.3%) was the most common complication, followed by pulmonary complications (11.4%) and fistula recurrence (6.8%). Only sepsis and multiorgan failure rates significantly differed between survivors and nonsurvivors (10.7% vs. 34.5%, *P* < 0.001; 0 vs. 9.5%, *P* = 0.001). Six patients (2.7%) underwent reintervention within 30 days; the rate did not differ between the two groups.

### 3.4. Long-Term Outcomes

The mean follow-up period was 8.6 months (interquartile range: 7 days–10.9 months). Kaplan–Meier survival analysis showed that the 1-year cumulative survival rate was 42.5 ± 3.8%. The overall fistula-related mortality rate was 47.0% (103/219). The most common causes of death were bleeding (54.9%) and infection (29.2%). Fourteen patients (6.4%) underwent unplanned reintervention after 30 days; the rate did not differ between the two groups.

**Figure 2:**
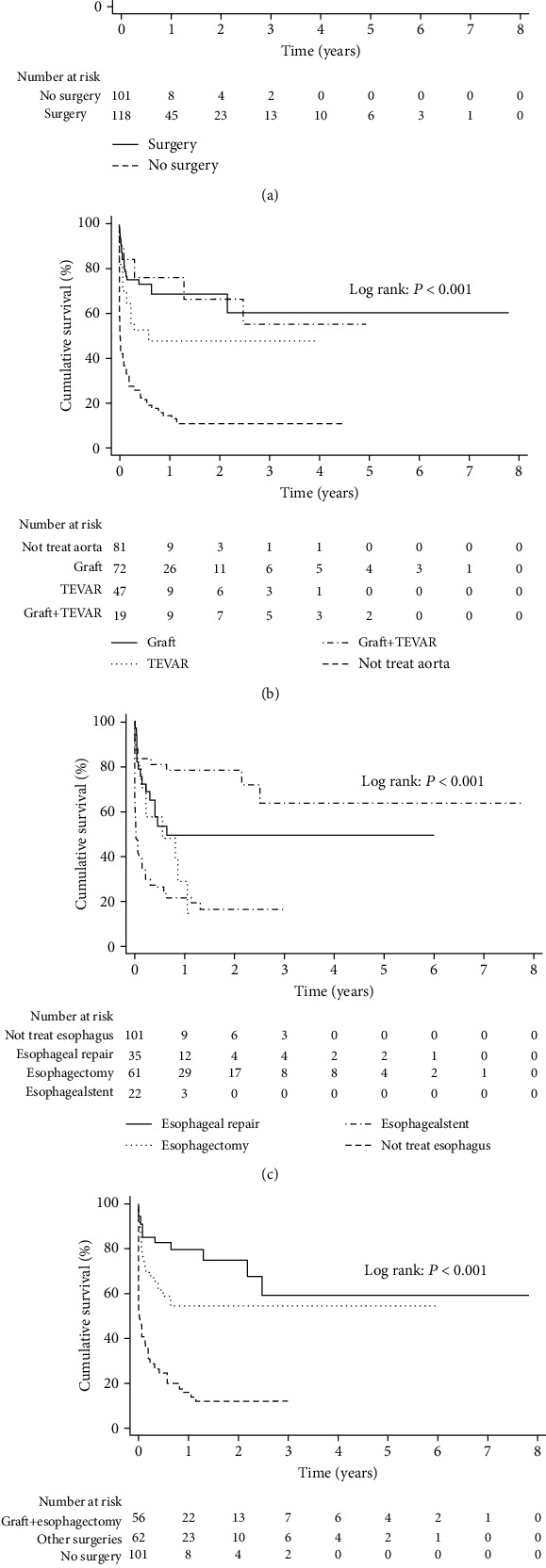
Overall survival of patients with aortoesophageal fistula (AEF). (a) Patients with AEF who underwent surgery for aortic or esophageal lesions had a significantly better prognosis compared to nonsurgical patients (*P* < 0.001). (b) Patients who were underwent graft replacement or graft replacement combined with TEVAR had a better prognosis than patients who received no treatment for aortic lesions (both *P* < 0.001). (c) Patients who underwent esophagectomy had better long-term survival compared to patients who underwent esophageal repair, esophageal stent, or no treatment (*P* = 0.040, *P* = 0.001, and *P* < 0.001, respectively). (d) Survival analysis confirmed better long-term survival of patients who underwent aortic graft replacement or esophagectomy compared to those who received other surgeries or treatments (*P* = 0.043, *P* < 0.001).

The overall survival rate of patients with AEF who underwent surgery for aortic or esophageal lesions was significantly higher than that of nonsurgical patients (*P* < 0.001, Figure 2). Concerning aortic therapy, a better prognosis was observed in patients treated with graft replacement than in patients treated with TEVAR (*P* = 0.047); however, no difference was found between the graft replacement plus TEVAR combination treatment and any single treatment (both *P* > 0.05). The worst prognosis was found among patients who received no treatment for aortic lesions (*P* < 0.001). Regarding esophageal therapy, patients who underwent esophagectomy had better long-term survival compared to patients who underwent esophageal repair, esophageal stent, or no treatment (*P* = 0.040, *P* = 0.001, and *P* < 0.001). No difference in the survival rate was found between patients who underwent esophageal repair and those who underwent esophageal stent (*P* = 0.276); both of these groups had better survival rates than patients who received no treatment (*P* < 0.001, *P* = 0.024). Better long-term survival was observed in patients who underwent aortic graft replacement or esophagectomy compared to those who received other surgeries or treatments (*P* = 0.043, *P* < 0.001).

### 3.5. Prognostic Factors

Univariable regression analysis showed that the odds of death increased with age and aortic rupture (*P* = 0.001, *P* = 0.012, Table 5) and decreased with the graft replacement plus TEVAR combination treatment (*P* = 0.042); however, these factors were not significant in a multivariable regression analysis (*P* = 0.587, *P* = 0.064, *P* = 0.189). Contrast leak in CT and aneurysm size were also associated with mortality (*P* = 0.018, *P* = 0.005) but were not included in the multivariable Cox regression analysis due to the lack of information in more than 15% of patients with AEF. Significant positive correlations of hemorrhagic shock, sepsis, multiorgan failure, and conservative treatment with mortality were found in multivariable regression analysis (*P* = 0.004, *P* = 0.015, *P* = 0.003, and *P* < 0.001), while aortic graft replacement and esophagectomy combination treatment was associated with significantly lower mortality (*P* = 0.017).

## 4. Discussion

AEF is a rare cause of upper gastrointestinal bleeding classically characterized by Chiari's triad [6], consisting of chest pain and sentinel hemorrhage followed by fatal exsanguination after an asymptomatic interval. In our study, hemorrhagic shock was the only initial presentation that was more frequently observed in patients with fatal AEF (*P* < 0.001); moreover, it was associated with mortality in multivariable analysis (*P* = 0.004). These findings were consistent with the results of prior studies, which also reported that hemodynamic shock was a risk factor for in-hospital mortality [7, 8]. Other studies reported that ectopic gas in the mediastina, a typical computed tomography finding of AEF [9], was associated with mortality; however, this was not confirmed in our study. And we found contrast leak in CT was associated with mortality in univariable analyses (*P* = 0.018). Aneurysm size also differed between survivors and nonsurvivors (*P* = 0.015) and was significantly associated with mortality in univariable Cox analysis (*P* = 0.005). Large thoracic aneurysms directly compress the esophagus and cause dysphagia, which may lead to aortoesophageal or aortobronchial fistulization [10–12]. Czerny et al. reported that aneurysm size was related to the development of post-TEVAR AEF due to mechanical compression and secondary erosion [3, 13]; esophageal ischemia and mediastinal infection were cited as potential causes. Notably, there was no significant difference in aneurysm size between patients with primary and secondary AEF.

Although mycotic AA accounts for 7.8% of total AEF patients, positive blood or tissue cultures were reported in up to 27.9% of AEF patients, dominated by *Staphylococcus aureus*, *Streptococcus* species, and *Candida* species. No significant differences were found in AEF types (primary or secondary AEF) between survivors and nonsurvivors; but for mycotic AA, primary AEF took a larger proportion in survivors while secondary AEF was just the opposite (*P* = 0.015). These findings emphasized the importance of broad-spectrum antibiotic or even antifungal therapy, especially for secondary AEF.

The overall in-hospital mortality rate was 42.0%; complications were observed in 38.4% of patients with AEF. Sepsis was the most common complication and was significantly correlated with mortality rate (*P* = 0.015), which emphasizes the role of infection control in the treatment of AEF [14]. Canaud et al. reported that use of broad-spectrum antibiotics for longer than 4 weeks in patients with AEF reduced the mortality rate [15]; in our study, 47.8% of patients with a follow-up period longer than 4 weeks received prolonged antibiotic treatment, but no significant difference was found between survivors and nonsurvivors (*P* = 0.102). Considering the risk of infection, broad-spectrum antibiotics should be recommended for a certain period; however, lifelong oral administration remains controversial [16, 17]. Other conservative treatments include proton pump inhibitors and jejunal nutrition [18]. Patients receiving such therapy are typically unable to undergo other treatments (especially surgery) due to their poor general condition and multiple comorbidities [19], and the outcome is almost invariably fatal for patients with AEF (55/57, 96.5%).

Surgery for AEF is performed to repair the fistula and control bleeding and infection [20, 21]. The approaches include extra-anatomical bypass or in situ replacement with grafts, as well as repair or resection of esophageal fistula with primary or secondary reconstruction [1, 22, 23]. In our study, the overall survival rate of patients with AEF who underwent surgery either for aortic or esophageal lesions was significantly higher compared to that of nonsurgical patients with AEF (*P* < 0.001). Moulakakis et al. reported that surgical treatment improved the prognosis of patients with infected thoracic aortic endografts with aortic fistulas [24]. Furthermore, aortic graft replacement and esophagectomy combination therapy achieved a better prognosis than other surgeries (*P* = 0.043), in association with a significantly lower mortality rate (*P* = 0.017). The 1-year survival rate reached 79.0%, compared to 54.0% for other surgeries. A recent questionnaire survey also demonstrated that aortic replacement combined with esophagectomy improved the survival rate of post-TEVAR patients [25]. Other researchers advocated this strategy as the only radical treatment for AEF that achieved high success rates in patients with post-TEVAR AEF [26, 27]. These results indicated the importance of addressing sources of bleeding and infection [28]. However, the mortality rate of this approach reached 18.4% in our study and was associated with considerable complications that required attention [29]. Both Dacron graft and cryopreserved aortic allograft were common choices, and cryopreserved aortic allograft was considered to be resistant against infection with a relative low reinfection rate when treated with aortic infections [30]. But its availability, whether match the patients' aortic shape, and delay of transportation all might limit its application in emergency surgery [31]. So antibiotic-soaked prosthetic grafts covered with omental flaps would be an effective alternative, especially when most of the AEF required emergency operations. Esophageal repair of the fistula was another option, especially when esophageal defects were minimal without gross contamination [32, 33].

TEVAR for patients with AEF has the advantages of rapid control of bleeding and minimal invasiveness [34], especially for patients with severe comorbidities [35, 36]. Although a better prognosis was observed in patients with AEF treated with TEVAR compared to those treated with conservative therapy (*P* < 0.001), no correlation was found between TEVAR and mortality in Cox regression (*P* > 0.05). TEVAR can be recommended only as an emergency bridging therapy before surgery [37, 38]. Radical surgery should be performed as soon as possible once the condition is stable because TEVAR can control bleeding but cannot debride infection [39, 40]. Sattah et al. recommended TEVAR as first-line treatment before aortic surgery for all patients with AEF, even those with stable hemodynamics [41]. However, we found that additional TEVAR with graft replacement was not superior to graft replacement alone (*P* > 0.05).

Endoscopic self-expanding esophageal stents are typically used for the palliation of malignant esophageal stricture; they can be helpful to control bleeding in esophageal fistula and prevent the entry of esophageal contents into the fistula [42]. An esophageal stent alone or combined with TEVAR could be a palliative option for patients who are unable to tolerate radical surgery [43, 44]. Although the longest reported poststent survival has been 8 months [42], the prognosis is typically unfavorable [18].

This study had several limitations. First, the data were obtained from case reports and case series published by different researchers, and the lack of uniformity of reporting items could have affected the results. Second, publication bias might have existed because physicians are inclined to publish positive results and successful patient outcomes. A randomized prospective study was impossible because of the low incidence of AEF, so our findings can only be used for informational purposes.

## 5. Conclusions

In this study, we identified the risk factors for death in patients with AEF related to aortic aneurysm and dissection. Hemorrhagic shock, sepsis, and multiorgan failure were risk factors for death in patients with AEF. Additionally, conservative treatment was associated with a higher mortality rate, while combined aortic graft replacement and esophagectomy improved the prognosis. We hope that these findings can improve the prognosis of patients with AEF.

## Figures and Tables

**Table 1:** Demographic, clinical, radiographic, and endoscopic findings of patients with AEF.

	Total *n* = 219	Survivors *n* = 103	Nonsurvivors *n* = 116	*P*
Male sex	63.1% (137/217)	62.7% (64/102)	63.5% (73/115)	1.000
Mean age (years)	65.8 ± 13.5	62.4 ± 13.2	68.9 ± 13.0	<0.001
Type of AEF				0.077
Primary	44.7% (98/219)	51.5% (53/103)	38.8% (45/116)	
Secondary	55.3% (121/219)	48.5% (50/103)	61.2% (71/116)	
Comorbidity				
Hypertension	34.5% (48/139)	28.8% (19/66)	39.7% (29/73)	0.212
Other arterial diseases	24.5% (34/139)	21.2% (14/66)	27.4% (20/73)	0.434
Coronary heart disease	13.7% (19/139)	16.7% (11/66)	11.0% (8/73)	0.459
COPD	12.9% (18/139)	12.1% (8/66)	13.7% (10/73)	0.806
Renal disease	12.2% (17/139)	9.1% (6/66)	15.1% (11/73)	0.312
Diabetes	9.4% (13/139)	4.5% (3/66)	13.7% (10/73)	0.082
Hyperlipidemia	7.2% (10/139)	3.0% (2/66)	11.0% (8/73)	0.101
Clinical presentation				
Hemorrhage	86.3% (183/212)	86.0% (86/100)	86.6% (97/112)	1.000
Hemorrhagic shock	50.5% (105/208)	37.1% (36/97)	62.2% (69/111)	<0.001
Chest/abdominal/back pain	38.3% (79/206)	39.6% (38/96)	37.3% (41/110)	0.775
Dysphagia	11.7% (24/206)	12.5% (12/96)	10.9% (12/110)	0.829
Fever	25.2% (52/206)	27.1% (26/96)	23.6% (26/110)	0.631
Chiari's triad	20.0% (41/205)	15.8% (15/95)	23.6% (26/110)	0.220
Elevated inflammatory markers	73.3% (63/86)	77.1% (27/35)	70.6% (36/51)	0.622
Positive cultures	27.9% (61/219)	26.2% (27/103)	29.3% (34/116)	0.652
Mycotic AA	7.8% (17/219)	6.8% (7/103)	8.6% (10/116)	0.801
Imaging features				
Mediastinal air bubbles in CT	39.9% (71/178)	35.2% (32/91)	44.8% (39/87)	0.221
Contrast leak in CT	11.2% (20/178)	8.8% (8/91)	13.8% (12/87)	0.346
Contrast leak in angiography	36.7% (11/30)	44.4% (8/18)	25.0% (3/12)	0.442
Contrast leak in esophagogram	73.7% (14/19)	60.0% (6/10)	88.9% (8/9)	0.303
Endoscopic features				
Tumor-like submucosal mass	21.9% (35/160)	22.1% (17/77)	21.7% (18/83)	1.000
Blood clot	40.0% (64/160)	41.6% (32/77)	38.6% (32/83)	0.748
Mucosa necrosis	42.5% (68/160)	45.5% (35/77)	39.8% (33/83)	0.523
Pulsatile arterial bleeding	7.5% (12/160)	7.8% (6/77)	7.2% (6/83)	1.000
Visible aortic wall or graft	23.1% (37/160)	22.1% (17/77)	24.1% (20/83)	0.852
Type of aortic lesion				1.000
Aneurysm	84.5% (185/219)	84.5% (87/103)	84.5% (98/116)	
Dissection	15.5% (34/219)	15.5% (16/103)	15.5% (18/116)	
Location of fistula				
Descending aorta	89.0% (195/219)	90.3% (93/103)	87.9% (102/116)	0.667
Aortic arch	17.4% (38/219)	15.5% (16/103)	19.0% (22/116)	0.593
Ascending aorta	2.7% (6/219)	3.9% (4/103)	1.7% (2/116)	0.424
Upper esophagus	19.4% (24/124)	18.6% (11/59)	20.0% (13/65)	0.743
Middle esophagus	66.1% (82/124)	62.7% (37/59)	69.2% (45/65)	0.455
Lower esophagus	16.1% (20/124)	18.6% (11/59)	13.8% (9/65)	0.626
Fistula size (cm)	2.2 ± 1.8 (60/219)	2.0 ± 1.6 (30/60)	2.4 ± 2.0 (30/60)	0.391
Aneurysm size (cm)	6.7 ± 3.0 (72/219)	5.8 ± 2.6 (33/72)	7.5 ± 3.2 (39/72)	0.015
Complications	38.4% (84/219)	28.2% (29/103)	47.4% (55/116)	0.004
Sepsis	23.3% (51/219)	10.7% (11/103)	34.5% (40/116)	<0.001
Multiorgan failure	5.0% (11/219)	0	9.5% (11/116)	0.001
Pulmonary complications	11.4% (25/219)	13.6% (14/103)	9.5% (11/116)	0.397
Renal failure	3.2% (7/219)	1.9% (2/103)	4.3% (5/116)	0.451
Neural complications	4.1% (9/219)	2.9% (3/103)	5.2% (6/116)	0.506
Aortic rupture	2.3% (5/219)	0	4.3% (5/116)	0.062
Fistula recurrence	6.8% (15/219)	6.8% (7/103)	6.9% (8/116)	1.000
Re-interventions within 30 d	2.7% (6/219)	2.9% (3/103)	2.6% (3/116)	1.000
Reinterventions unplanned after 30 d	6.4% (14/219)	7.8% (8/103)	5.2% (6/116)	0.582

AA: aortic aneurysm; AEF: aortoesophageal fistula; CT: computed tomography; SD: standard deviation.

Data are counts, percentages, means ± standard deviations or medians. Numbers of patients with available data are shown in parentheses.

**Table 2:** Survival data for mycotic AA in AEF patients.

	Total *n* = 17	Survivors *n* = 7	Nonsurvivors *n* = 10	*P*
Type of AEFs				0.015
Primary AEFs	47.1% (8/17)	85.7% (6/7)	20.0% (2/10)	
Secondary AEFs	52.9% (9/17)	14.3% (1/7)	80.0% (8/10)	

AA: aortic aneurysm; AEF: aortoesophageal fistula.

**Table 3:** Treatments and outcomes of patients with AEF.

	Total *n* = 219	Survivors *n* = 103	Nonsurvivors *n* = 116	*P*
Treatment for aortic lesions				
Aortic repair	2.3% (5/219)	1.9% (2/103)	2.6% (3/116)	1.000
Aortic graft	32.9% (72/219)	49.5% (51/103)	18.1% (21/116)	<0.001
TEVAR	21.5% (47/219)	26.2% (27/103)	17.2% (20/116)	0.138
Aortic graft+TEVAR	8.7% (19/219)	12.6% (13/103)	5.2% (6/116)	0.057
No treatment	34.7% (76/219)	9.7% (10/103)	56.9% (66/116)	<0.001
Treatment for esophageal lesions				
Esophageal repair	16.0% (35/219)	19.4% (20/103)	12.9% (15/116)	0.202
Esophagectomy	27.9% (61/219)	45.6% (47/103)	12.1% (14/116)	<0.001
Esophageal stents	10.0% (22/219)	9.7% (10/103)	10.3% (12/116)	1.000
Esophageal combination treatments	5.0% (11/219)	7.8% (8/103)	2.6% (3/116)	0.120
No treatment	41.1% (90/219)	17.5% (18/103)	62.1% (72/116)	<0.001
Combination of therapy				
Conservative treatment	26.0% (57/219)	1.9% (2/103)	47.4% (55/116)	<0.001
Aortic repair±esophageal surgery	2.3% (5/219)	1.9% (2/103)	2.6% (3/116)	1.000
TEVAR	11.9% (26/219)	12.6% (13/103)	11.2% (13/116)	0.835
TEVAR+esophageal repair	2.3% (5/219)	2.9% (3/103)	1.7% (2/116)	0.668
TEVAR+esophagectomy	1.8% (4/219)	2.9% (3/103)	0.9% (1/116)	0.344
TEVAR+esophageal stent	4.6% (10/219)	6.8% (7/103)	2.6% (3/116)	0.196
Aortic graft	2.3% (5/219)	1.9% (2/103)	2.6% (3/116)	1.000
Aortic graft+esophageal repair	10.0% (22/219)	12.6% (13/103)	7.8% (9/116)	0.265
Aortic graft+esophagectomy	17.4% (38/219)	30.1% (31/103)	6.0% (7/116)	<0.001
Aortic graft+esophageal stent	0.9% (2/219)	1.0% (1/103)	0.9% (1/116)	1.000
TEVAR+aortic graft±esophageal treatments	8.7% (19/219)	12.6% (13/103)	5.2% (6/116)	0.057
TEVAR/aortic graft+esophageal treatments	3.2% (7/219)	4.9% (5/103)	1.7% (2/116)	0.258
Esophageal repair	1.8% (4/219)	1.0% (1/103)	2.6% (3/116)	0.624
Esophagectomy	3.2% (7/219)	5.8% (6/103)	0.9% (1/116)	0.053
Esophageal stent	3.7% (8/219)	1.0% (1/103)	6.0% (7/116)	0.069
Antibiotics (>4 weeks)	47.8% (65/136)	53.3% (48/90)	37.0% (17/46)	0.102

AEF: aortoesophageal fistula; TEVAR: thoracic endovascular aortic repair.

Data are percentages. Numbers of patients with available data are shown in parentheses.

**Table 4:** Summary of grafts used for aortic replacement.

	Total	Survivors	Nonsurvivors	*P*
Prosthetic grafts	27.6% (59/214)	43.0% (43/100)	14.0% (16/114)	<0.001
PTFE grafts	1.0% (2/201)	2.2% (2/92)	0	0.208
Dacron grafts	23.9% (48/201)	39.1% (36/92)	11.0% (12/109)	<0.001
Cryopreserved aortic allograft	10.3% (22/214)	15.0% (15/100)	6.1% (7/114)	0.042
Pericardial tube	0.5% (1/214)	1.0% (1/100)	0	0.467

PTFE: polytetrafluoroethylene.

**Table 5:** Cox regression analyses.

	Univariable Cox regression			Multivariable Cox regression		
	HR	95% CI	*P*	HR	95% CI	*P*
Age	1.027	1.011-1.043	0.001	1.004	0.988-1.021	0.587
Hemorrhagic shock	2.124	1.444-3.123	<0.001	1.824	1.217-2.735	0.004
Contrast leak in CT†	2.102	1.134-3.897	0.018			
Aneurysm size†	1.155	1.044-1.279	0.005			
Sepsis	1.900	1.291-2.795	0.001	1.714	1.112-2.641	0.015
Multiorgan failure	3.544	1.872-6.710	<0.001	3.060	1.470-6.368	0.003
Aortic rupture	3.186	1.289-7.786	0.012	2.410	0.949-6.117	0.064
Conservative treatment	6.570	4.459-9.681	<0.001	5.257	3.405-8.116	<0.001
Aortic graft+esophagectomy	0.214	0.099-0.460	<0.001	0.319	0.125-0.813	0.017
TEVAR+aortic graft±esophageal treatments	0.424	0.186-0.968	0.042	0.493	0.172-1.415	0.189

AEF: aortoesophageal fistula; TEVAR: thoracic endovascular aortic repair; CT: computed tomography; HR: hazard ratio; CI: confidence interval.

†Contrast leak in CT and aneurysm size were excluded from multivariate regression analysis due to the lack of information in more than 15% of patients with AEF.
