# Supplementary material for: Risk Factors for Mortality in Patients with Aortoesophageal Fistula Related to Aortic Lesions
Source: Gastroenterol Res Pract. 2020 Sep 17;2020:4850287. doi: 10.1155/2020/4850287 (PMC7519457; doi:10.1155/2020/4850287)
Supplement: Supplementary Materials — Detailed information on the included articles is provided in the supplementary file. [file 4850287.f1.docx]

[1] Madan AK, Santora TA, Disesa VJ. Extra-anatomic bypass grafting for aortoesophageal fistula: a logical operation. *J Vasc Surg* 2000; **32**: 1030-3.

[2] Mehta VK, Lafaro RJ, De Vincenzo S. Successful management of an aneurysmal aortoesophageal fistula. *J Cardiovasc Surg* 2000; **41**: 721-3.

[3] Reardon MJ, Brewer RJ, LeMaire SA, Baldwin JC, Safi HJ. Surgical management of primary aortoesophageal fistula secondary to thoracic aneurysm. *Ann Thorac Surg* 2000; **69**: 967-70.

[4] Bond SE, McGuinness CL, Reidy JF, Taylor PR. Repair of secondary aortoesophageal fistula by endoluminal stent-grafting. *J Endovasc Ther* 2001; **8**: 597-601.

[5] Iguchi A, Miyazaki S, Akimoto H, Khalil K, Safi HJ, Estrera AL. Successful management of secondary aortoesophageal fistula with graft infection. *Thorac Cardiovasc Surg* 2001; **49**: 126-8.

[6] Lau H. Dysphagia aortica: harbinger of aortoesophageal fistula? *Surgery* 2001; **129**: 763-4.

[7] Patel MA, Schmoker JD, Moses PL, Anees R, D'Agostino R. Mycotic arch aneurysm and aortoesophageal fistula in a patient with melioidosis. *Ann Thorac Surg* 2001; **71**: 1363-5.

[8] Tseng LJ, Jao YT, Mo LR. Endoscopic images of aortoesophageal fistula. *Gastrointest Endosc* 2001; **53**: 631.

[9] D'Ancona G, Dagenais F, Bauset R. Endoluminal stenting of the aorta as treatment of aortoesophageal fistula due to primary aortic disease. *Tex Heart Inst J* 2002; **29**: 216-7.

[10] Eloubeidi MA, Borlaza RF, Canon CL, Wilcox C Mel. Aorta-esophageal fistula. *Gastrointest Endosc* 2002; 56: 417.

[11] Pellizzari N, Reimers B, Sacca S, et al. Percutaneous endovascular stent-graft placement for the treatment of acute rupture of an aneurysm of the thoracic aorta. *Ital Heart J* 2002; **3**: 427-30.

[12] Shiraishi S, Watarida S, Matsubayashi K, Matsubayashi, K, Motoishi M, Satsu T.

Successful management of an aortoesophageal fistula resulting from an aneurysm of the thoracic aorta with a covered stent. *J Cardiovasc Surg* 2002; **43**: 95-8.

[13] Taniguchi I, Takemoto N, Yamaga T, Morimoto K, Miyasaka S, Suda T. Primary aortoesophageal fistula secondary to thoracic aneurysm. Successful surgical treatment by extra-anatomic bypass grafting. *Jpn J Thorac Cardiovasc Surg* 2002; **50**: 263-7.

[14] Van Doorn RC, Reekers J, de Mol BA, Obertop H, Balm R. Aortoesophageal fistula secondary to mycotic thoracic aortic aneurysm: endovascular repair and transhiatal esophagectomy. *J Endovasc Ther* 2002; **9**: 212-7.

[15] Yasuda F, Shimono T, Tonouchi H, Shimpo H, Yada I. Successful repair of an aortoesophageal fistula with aneurysm from esophageal diverticulum. *Ann Thorac Surg* 2002; **73**: 637-9.

[16] Yuan WH, Chen JD, Tiu CM, et al. Mycotic aneurysm of thoracic aorta with aortoesophageal fistula mimicking the esophageal tumor with bleeding－a case report. *Kaohsiung J Med Sci* 2002; **18**: 248-52.

[17] Born C, Forster A, Rock C, Pfeifer KJ, Rieger J, Reiser M. A case of an upper gastrointestinal bleeding due to a ruptured dissection of a right aortic arch. *Cardiovasc Intervent Radiol* 2003; **26**: 506-9.

[18] Hance KA, Hsu J, Eskew T, Hermreck AS. Secondary aortoesophageal fistula after endoluminal exclusion because of thoracic aortic transection. *J Vasc Surg* 2003; **37**: 886-8.

[19] Kieffer E, Chiche L, Gomes D. Aortoesophageal fistula: value of in situ aortic allograft replacement. *Ann Surg* 2003; **238**: 283-90.

[20] Lee EB, Lee SC, Cho JY, Lee JT. Surgery for concomitant aortoesophageal and aortobronchial fistula in tuberculous aortitis. *Interact Cardiovasc Thorac Surg* 2003; **2**: 234-6.

[21] Reddi A, Chetty R. Primary aorto-esophageal fistula due to Takayasu's aortitis. *Cardiovasc Pathol* 2003; **12**: 112-4.

[22] Unosawa S, Akiyama K, Nakata K, et al. Successful surgical treatment for an aortoesophageal fistula due to a descending aortic aneurysm. *Ann Thorac Cardiovasc Surg* 2003; **9**: 257-60.

[23] Eggebrecht H, Baumgart D, Radecke K, et al. Aortoesophageal fistula secondary to stent-graft repair of the thoracic aorta. *J Endovasc Ther* 2004; **11**: 161-7.

[24] Flores J, Shiiya N, Kunihara T, Yoshimoto K, Yasuda K. Aortoesophageal fistula: alternatives of treatment case report and literature review. *Ann Thorac Cardiovasc* Surg 2004; **10**: 241-6.

[25] Martin M, Steele S, Mullenix P, Haque M, Andersen C. Endoscopic diagnosis of a clinically silent aortoesophageal fistula: case report and review of the literature. *Ann Vasc Surg* 2004; **18**: 352-6.

[26] Nishibe T, Koizumi J, Kudo F, Miyazaki K, Nishibe M, Yasuda K. Successful endovascular stent-graft treatment for an aortoesophageal fistula caused by a descending thoracic aortic aneurysm: report of a case. *Surg Today* 2004; **34**: 529-31.

[27] Tokuda Y, Matsumoto M, Sugita T, et al. Successful repair of an aortoesophageal fistula caused by a thoracic aortic aneurysm: report of a case. *Surg Today* 2004; **34**: 357-9.

[28] Ben Mansour el H, Bachet J, Tran D, Lamer C. Ruptured mycotic aortic pseudoaneurysm with concomitant aorto-tracheal and aorto-oesophageal fistulas. *Eur J Cardiothorac Surg* 2005; 27: 711.

[29] Czerny M, Zimpfer D, Fleck T, et al. Successful treatment of an aortoesophageal fistula after emergency endovascular thoracic aortic stent-graft placement. *Ann Thorac Surg* 2005; **80**: 1117-20.

[30] Eren E, Keles C, Toker ME, et al. Surgical treatment of aortobronchial and aortoesophageal fistulae due to thoracic aortic aneurysm. *Tex Heart Inst J* 2005; **32**: 522-8.

[31] González-Fajardo JA, Gutiérrez V, Martín-Pedrosa M, Del Rio L, Carrera S, Vaquero C. Endovascular repair in the presence of aortic infection. *Ann Vasc Surg* 2005; **19**: 94-8.

[32] Kimura N, Kawahito K, Murata S, Yamaguchi A, Adachi H, Ino T. Aortobronchial fistula resulting from a mycotic pseudoaneurysm after treatment of an aortoesophageal fistula due to a thoracic aortic aneurysm. *Jpn J Thorac Cardiovasc Surg* 2005; **53**: 619-23.

[33] Luckraz H, Kitchlu S, Youhana A. Aortoesophageal fistula as a late complication of aortic transection. *J Thorac Cardiovasc Surg* 2005; **129**: 458-9.

[34] Pirard L, Creemers E, Van Damme H, Laurent S, Honore P, Limet R. In situ aortic allograft insertion to repair a primary aortoesophageal fistula due to thoracic aortic aneurysm. *J Vasc Surg* 2005; **42**: 1213-7.

[35] Porcu P, Chavanon O, Sessa C, Thony F, Aubert A, Blin D. Esophageal fistula after endovascular treatment in a type B aortic dissection of the descending thoracic aorta. *J Vasc Surg* 2005; **41**: 708-11.

[36] Raghavendran K, Cherr GS, Ford PF, Burkhard PG, Bell-Thomson J. Successful management of concomitant aortoesophageal and aortotracheal fistulae secondary to a thoracic aortic aneurysm: case report and review of literature. *J Vasc Surg* 2005; **42**: 1218-20.

[37] Contini S, Corrente V, Nervi G, Franzè A, Scarpignato C. Dysphagia aortica: a neglected symptom of aortoesophageal fistula. *Dig Liver Dis* 2006; **38**: 51-4.

[38] Kitayama J, Morota T, Kaisaki S, et al. Complete coverage of in situ aortograft by total omental pedicle flap as the most reliable treatment of aortoesophageal fistula. *Am J Surg* 2006; **192**: 130-4.

[39] Zisis C, Exarchos D, Kotsifas K, Argiriou M, Portinos A, Bellenis I. Aortic pseudoaneurysm rupture into the oesophagus. *Asian Cardiovasc Thorac Ann* 2006; **14**: 441-2.

[40] Iwaki H, Kuraoka S, Tatebe S, et al. Pneumatic aorta: aortoesophageal fistula due to chronic aortic dissection. *J Nippon Med Sch* 2007; **74**: 90-1.

[41] Martens K, De Mey J, Everaert H, Delvaux G, van den Brande P. Aortoesophageal fistula following endovascular exclusion of a thoracic aneurysm. *Int Angiol* 2007; **26**: 292-6.

[42] Miyatani H, Yoshida Y. Usefulness of endoscopic ultrasonography with a microprobe in the diagnosis of aortoesophageal fistula. *J Med Ultrason* 2007; **34**: 205-7.

[43] Riesenman PJ, Farber MA, Mauro MA, Selzman CH, Feins RH. Aortoesophageal fistula after thoracic endovascular aortic repair and transthoracic embolization. *J Vasc Surg* 2007; **46**: 789-91.

[44] Topel I, Stehr A, Steinbauer MG, Piso P, Schlitt HJ, Kasprzak PM. Surgical strategy in aortoesophageal fistulae: endovascular stentgrafts and in situ repair of the aorta with cryopreserved homografts. *Ann Surg* 2007; **246**: 853-9.

[45] Xia M, Guo JZ, Zhan Q, Yan J. Aortoesophageal fistula caused by descending aortic pseudoaneurysm: one case report. *Chin Med J* 2007; **120**: 2149-50.

[46] Akaraviputh T, Sriprayoon T, Prachayakul V, Sakiyalak P. Endoscopic diagnosis of secondary aortoesophageal fistula. *Endoscopy* 2008; **40 (Suppl 2)**: E90.

[47] Ang TL, Lim K, Kwek A, Teo EK, Fock KM. A rare case of upper GI bleeding: esophageal rupture associated with thoracic aortic aneurysm. *Gastrointest Endosc* 2008; **67**: 151-2.

[48] Barcellos Cda S, Azambuja PC, Momolli MK, et al. Aortic aneurysm rupture into the esophagus. *Arq Bras Cardiol* 2008; **91**: e61-3.

[49] Civilini E, Bertoglio L, Melissano G, Chiesa, R. Aortic and esophageal endografting for secondary aortoenteric fistula. *Eur J Vasc Endovasc Surg* 2008; **36**: 297-9.

[50] Girdauskas E, Falk V, Kuntze T, et al. Secondary surgical procedures after endovascular stent grafting of the thoracic aorta: Successful approaches to a challenging clinical problem. *J Thorac Cardiovasc Surg* 2008; **136**: 1289-94.

[51] Inoue T, Nishino T, Peng YF, Saga T. Successful one-stage operation of aortoesophageal fistula from thoracic aneurysm using a rifampicin-soaked synthetic graft. *Interact Cardiovasc Thorac Surg* 2008; **7**: 322-4.

[52] Kawamoto S, Saiki Y, Oda K, et al. Successful management of esophagoparaprosthetic fistula after aortic surgery. *Ann Thorac Surg* 2008; **85**: 1449-51.

[53] Lin CS, Tung F, Yeh HZ, et al. Aortoesophageal fistula with a history of graft treatment for thoracic aortic aneurysm. *J Chin Med Assoc* 2008; **71**: 100-2.

[54] Lin IT, Shih SC, Wang TE, Chang CS, Lin CW. A rare etiology of hematemesis: esophageal erosive ulcer bleeding caused by aortic aneurysm compression. *Gastrointest Endosc* 2008; **68**: 148-9.

[55] Saha P, Burnand KG, Patel SD, Waltham, M. Complications after endoluminal stent grafting of a thoracic mycotic aneurysm. *Circulation* 2008; **117**: 3157-9.

[56] Zuber-Jerger I, Hempel U, Rockmann F, Klebl F. Temporary stent placement in 2 cases of aortoesophageal fistula. *Gastrointest Endosc* 2008; **68**: 599-2.

[57] Alrubaiy L, Sutton J, Ahmed W. Dissecting thoracic aortic aneurysm presenting with haematemesis. *BMJ Case Rep* 2009; **2009**.

[58] Chan YC, Ting AC, Law S, Cheng SW. Secondary infection of a pre-existing thoracic aortic aneurysm by iatrogenic oesophageal perforation with aorta-oesophageal fistula formation. *Eur J Cardiothorac Surg* 2009; **35**: 365-7.

[59] Christensen JD, Heyneman LE. Case of the season: aortoesophageal fistula complicating thoracic aortic aneurysm stent graft repair. *Semin Roentgenol* 2009; **44**: 4-7.

[60] Eggebrecht H, Mehta RH, Dechene A, et al. Aortoesophageal fistula after thoracic aortic stent-graft placement. A rare but catastrophic complication of a novel emerging technique. *JACC Cardiovasc Interv* 2009; **2**: 570-6.

[61] Fukunaga N, Matsueda T, Osumi M, et al. Unexpectedly large aortoesophageal fistula inconsistent with CT imaging due to the thrombus working as the tamponade. *J Cardiol* 2009; **54**: 466-9.

[62] Grimminger P, Vallbohmer D, Bludau M, Brabender J, Metzger R, Holscher AH. Successful management of esophageal perforation due to an aortic arch aneurysm replacement. *Dis Esophagus* 2009; **22**: 471-4.

[63] Huang SC, Lin TC, Tsan YT, Master SY. Catastrophic gastrointestinal bleeding caused by aortoesophageal fistula secondary to mycotic thoracic aortic aneurysm. *BMJ Case Rep* 2009; **2009**.

[64] Isasti G, Gómez-Doblas JJ, Olalla E. Aortoesophageal fistula: An uncommon complication after stent-graft repair of an aortic thoracic aneurysm. *Interact Cardiovasc Thorac Surg* 2009; **9**: 683-4.

[65] Jiao Y, Zong Y, Yu ZL, Yu YZ, Zhang ST. Aortoesophageal fistula: a case misdiagnosed as esophageal polyp. *World J Gastroenterol* 2009; **15**: 6007-9.

[66] Jonker FH, Heijmen R, Trimarchi S, Verhagen HJM, Moll FL, Muhs BE. Acute management of aortobronchial and aortoesophageal fistulas using thoracic endovascular aortic repair. *J Vasc Surg* 2009; **50**: 999-1004.

[67] Nakamura S, Mizuno K, Haruyama H, Kishino M, Konishi H, Shiratori K. Gastrointestinal: aorto-esophageal fistula. *J Gastroenterol Hepatol* 2009; **24**: 698.

[68] Prokakis C, Charoulis N, Tselikos D, Koletsis EN, Apostolakis E, Dougenis D. Primary aortoesophageal fistula due to thoracic aortic aneurysm: successful surgical treatment. *Tex Heart Inst J* 2009; **36**: 607-10.

[69] Torrado H, Ventura JL, Farrero E. Aortoesophageal fistula, a catastrophic complication soon after successful repair of an aortic dissection type A. *Eur Heart J* 2009; **30**: 32.

[70] Ambepitiya SG, Michiue T, Bessho Y, Kamikodai Y, Ishikawa T, Maeda H. An unusual presentation of thoracic aortic aneurysm rupturing into the esophagus: an autopsy case report. *Forensic Sci Med Pathol* 2010; **6**: 121-6.

[71] Chen JS, Chiu KM, Chu SH, Huang JH. Esophageal erosion after stent graft implantation for a Salmonella mycotic aneurysm. *Ann Thorac Surg* 2010; **90**: 672.

[72] Hyun S, Song HJ, Choi EK, et al. Life-threatening aortoesophageal fistula bleeding associated with mycotic aneurysm of descending thoracic aorta [Abstract]. *Digest Endosc* 2010; **22**: A39.

[73] Kim HW, Suh JH, Jo KH, Yoon JS. Concomitant aortoesophageal and aortobronchial fistula after endovascular aortic repair. *Ann Thorac Surg* 2010; **90**: 2062.

[74] Marone EM, Coppi G, Kahlberg A, Tshomba Y, Chiesa R. Combined endovascular and surgical treatment of primary aortoesophageal fistula. *Tex Heart Inst J* 2010; **37**: 722-4.

[75] Yavuz S, Agirbas H, Parlar H, et al. A catastrophic complicaton of endovascular stent-graft repair of ruptured descending aortic aneurysm. *Interact Cardiovasc Thorac Surg* 2010; **10**: S116.

[76] Albors J, Bahamonde JA, Sanchis JM, Boix R, Palmero J. Aortoesophageal fistula after thoracic stent grafting. *Asian Cardiovasc Thorac Ann* 2011; **19**: 352-6.

[77] Bakhshandeh AR, Salehi M, Radmehr H, Riahi GR. A case of aortoesophageal fistula. *Asian Cardiovasc Thorac Ann* 2011; **19**: 419-21.

[78] Bargiggia S, Parente F, Rossi G, Lorenzi G. Expulsion of vascular atheroma through aorto-esophageal fistula. *J Gastrointestin Liver Dis* 2011; **20**: 7.

[79] Ferrero E, Viazzo A, Ferri M, et al. Acute management of aortoesophageal fistula and tracheoesophageal fistula treated by thoracic endovascular aortic repair and esophageal endoprosthesis: a case misdiagnosed as esophageal cancer. *Ann Vasc Surg* 2011; **25**: 1142.e1-5.

[80] Gomes SIM, de Campos FPF, Martines BMR, Martines Jads, Tafner E, Maruta LM. Primary aortoesophageal fistula: a rare cause of acute upper gastrointestinal bleeding. *Autops Case Rep* 2011; **1**: 57-63.

[81] Gavens E, Zaidi Z, Al-Jundi W, Kumar P. Aortoesophageal fistula after endovascular aortic aneurysm repair of a mycotic thoracic aneurysm. *Int J Vasc Med* 2011; **2011**: 649592.

[82] Ishikawa M, Toyota N, Kakizawa H, Matsuura N, Hieda M, Awai K. Aortoesophageal fistula after stent-graft treatment of an aortic arch mycotic aneurysm: treatment with embolization and covered esophageal stent. *J Vasc Interv Radiol* 2011; **22**: 578-81.

[83] Kasai K, Ushio A, Tamura Y, et al. Conservative treatment of an aortoesophagial fistula after endovascular stent grafting for a thoracic aortic aneurysm. *Med Sci Monit* 2011; **17**: Cs39-42.

[84] Kritpracha B, Premprabha D, Sungsiri J, Tantarattanapong W, Rookkapan S, Juntarapatin P. Endovascular therapy for infected aortic aneurysms. *J Vasc Surg* 2011; **54**: 1259-65.

[85] Miller TN, Miller RA, Ziebarth J. Aortoesophageal fistula secondary to aortic dissection: case report and review. *S D Med* 2011; **64**: 129-30.

[86] Sager HB, Wellhoner P, Wermelt JA, Schunkert H, Kurowski V. Lethal hemorrhage caused by aortoesophageal fistula secondary to stent-graft repair of the thoracic aorta. *Cardiovasc Intervent Radiol* 2011; **34 (Suppl 2)**: S60-3.

[87] Tsai SM, Chen YY, Chin-Yuan Y, Lai W L. Closure of an aortoesophageal fistula with an esophageal stent and hemoclip. *Endoscopy* 2011; **43 (Suppl 2) UCTN**: E302-3.

[88] Tseng KC, Lin CW, Tan JW. Successful management of aortoesophageal fistula by combining endoscopic cyanoacrylate injection and endovascular stent grafting. *Endoscopy* 2011; **43 (Suppl 2) UCTN**: E135-6.

[89] Vasquez JC, Delarosa J, Leon JJ. Aortoesophageal fistula as a late complication of type B aortic dissection. *Vascular* 2011; **19**: 55-8.

[90] Yamanaka K, Nonaka M, Iwakura A, Asao Y. Repair of aortoesophageal fistula after total aortic arch grafting. *Interact Cardiovasc Thorac Surg* 2011; **12**: 655-6.

[91] Yavuz S, Kanko M, Ciftci E, Parlar H, Agirbas H, Berki T. Aortoesophageal fistula secondary to thoracic endovascular aortic repair of a descending aortic aneurysm rupture. *Heart Surg Forum* 2011; **14**: E249-51.

[92] Abdulaziz S, Abou-Shala N, Al-Sanouri I. A young patient with massive haematemesis. *BMJ Case Rep* 2012; **2012**.

[93] Amano M, Azuma T, Izumi C, et al. Aortic prosthetic graft infection accompanied with esophagomediastinal fistulas: a case report. *J Cardiol Cases* 2012; **6**: e51-4.

[94] Dołega-Kozierowski B, Sokratous K, Dyś K, et al. Aortoesophageal fistula as a complication of thoracic aorta aneurism stent grafting－a case report and literature review. *Pol J Radiol* 2012; **77**: 77-80.

[95] Hadzisejdic I, Mustac E, Krstulja M, Franjic N, Stimac D. Thoracic aortic aneurysm rupture into the esophagus. *Forensic Sci Med Pathol* 2012; **8**: 327-9.

[96] Lai WL, Li PC, Li ML. Is it possible to treat aorto-esophageal fistula with endovascular management? *Ann Thorac Cardiovasc Surg* 2012; **18**: 564-8.

[97] Lee SH, Song PS, Kim WS, Park KB, Choi SH. A case of stent graft infection coupled with aorto-esophageal fistula following thoracic endovascular aortic repair in a complex patient. *Korean Circ J* 2012; **42**: 366-8.

[98] Numan F, Gulsen F, Cantasdemir M, Solak S, Arbatli H. Percutaneous treatment of an infected aneurysmal sac secondary to aortoesophageal fistula with a history of stent-graft treatment for thoracic aortic aneurysm. *Cardiovasc Intervent Radiol* 2012; **35**: 690-4.

[99] Rispoli P, Bertoldo U, Oliaro A, et al. Two-stage treatment of a secondary aortoesophageal fistula after thoracic endovascular aneurysm repair. *J Cardiovasc Surg* 2012; **53**: 531-5.

[100] Saito A, Motomura N, Hattori O, et al. Outcome of surgical repair of aorto-eosophageal fistulas with cryopreserved aortic allografts. *Interact Cardiovasc Thorac Surg* 2012; **14**: 532-7.

[101] Vallabhajosyula P, Komlo C, Wallen T, Szeto WY. Two-stage surgical strategy for aortoesophageal fistula: emergent thoracic endovascular aortic repair followed by definitive open aortic and esophageal reconstruction. *J Thorac Cardiovasc Surg* 2012; **144**: 1266-8.

[102] Abdelsalam M, Westra K, Mumtaz M, Bachinsky W. Aortoesophageal fistula secondary to descending aortic pseudoaneurysm [Abstract]. *Chest* 2013; **144**: 93A.

[103] Byard RW. Lethal aorto-oesophageal fistula－characteristic features and aetiology. *J Forensic Leg Med* 2013; **20**: 164-8.

[104] Canaud L, Alric P, Gandet T, Ozdemir BA, Albat B, Marty-Ane C. Open surgical secondary procedures after thoracic endovascular aortic repair. *Eur J Vasc Endovasc Surg* 2013; **46**: 667-74.

[105] Chiba D, Hanabata N, Araki Y, et al. Aortoesophageal fistula after thoracic endovascular aortic repair diagnosed and followed with endoscopy. *Intern Med* 2013; **52**: 451-5.

[106] De Masi M, Amabile P, Bal L, Piquet P. Management of endograft infection coupled with aortoesophageal fistula: extra-anatomic aortic bypass and endograft explantation. *J Thorac Cardiovasc Surg* 2013; **146**: e11-3.

[107] Dorweiler B, Weigang E, Duenschede F, Pitton M, Dueber C, Vahl CF. Strategies for endovascular aortic repair in aortobronchial and aortoesophageal fistulas. *Thorac Cardiovasc Surg* 2013; **61**: 575-80.

[108] Fatimi SH, Malik AI, Ashfaq A, Murtaza N. Primary pseudoaneurysm of the descending aorta. *Indian J Surg* 2013; **75 (Suppl 1)**: 472-4.

[109] Göbölös L, Miskolczi S, Pousios D, et al. Management options for aorto-oesophageal fistula: Case histories and review of the literature. *Perfusion* 2013; **28**: 286-90.

[110] Hsu WF, Lin CC, Chang KM, Lee TH. Primary aortoesophageal fistula: a rare but fatal cause of upper gastrointestinal bleeding. *J Dig Dis* 2013; **14**: 676-8.

[111] Kay MD, Davies B, Patel K, Gourevitch D. Aorto-oesophageal fistula following TEVAR: an unusual cause of mediastinal air. *BMJ Case Rep* 2013; **2013**.

[112] Kobayashi K, Ohata T, Ueda H, Shichinohe T. Management of secondary aortoesophageal fistula without graft extraction. *J Thorac Cardiovasc Surg* 2013; **145**: e5-6.

[113] Munakata H, Yamanaka K, Okada K, Okita Y. Successful surgical treatment of aortoesophageal fistula after emergency thoracic endovascular aortic repair: aggressive debridement including esophageal resection and extended aortic replacement. *J Thorac Cardiovasc Surg* 2013; **146**: 235-7.

[114] Muradi A, Yamaguchi M, Kitagawa A, et al. Secondary aortoesophageal fistula after thoracic endovascular aortic repair for a huge aneurysm. *Diagn Interv Radiol* 2013; **19**: 81-4.

[115] Onodera M, Inoue Y, Fujino Y, Kikuchi S, Endo S. A case of secondary aortoesophageal fistula inserted a covered self-expanding esophageal stent to control gastrointestinal bleeding. *Case Rep Gastrointest Med* 2013; **2013**: 857135.

[116] Ozaki K, Sanada J, Ohtake H, Watanabe G, Matsui O. Successful thoracic endovascular aortic repair of an aortoesophageal fistula. *Vascular* 2013; **21**: 97-101.

[117] Shichinohe T, Wakasa S, Kubota S, et al. One-stage radical operation of aortoesophageal fistula－combination of VATS esophagectomy and open aortic surgery: report of a case. *Esophagus* 2013; **10**: 280-4.

[118] Sitt JCM, Paunipagar BK, Rasalkar DD. Aortoesophageal fistula complicating thoracic aorta stent-graft placement. *Hong Kong J Radiol* 2013; **16**: 61-4.

[119] Xu R, Wang T, Li D, et al. Surgical approach for the treatment of aortoesophageal fistula combined with dual aortic aneurysms: a case report. *J Cardiothorac Surg* 2013; **8**: 206.

[120] Andrade LC, Felix-Morais R, Gil-Agostinho A, Caseiro-Alves F. Aorto-oesophageal fistula treated with emergent thoracic endovascular repair. *BMJ Case Rep* 2014; **2014**.

[121] Ayyildiz T, Felix-Morais R, Gil-Agostinho A, Dolar E, Gur̈el S. A rare upper gastrointestinal system bleeding case: aortoesophageal fistula. *J Experim Clin Med* 2014; **31**:51-3.

[122] Dumfarth J, Dejaco H, Krapf C, et al. Aorto-esophageal fistula after thoracic endovascular aortic repair: successful open treatment. *Aorta* 2014; **2**:37-40.

[123] Kahlberg A, Tshomba Y, Marone EM, Castellano R, Melissano G, Chiesa R. Current results of a combined endovascular and open approach for the treatment of aortoesophageal and aortobronchial fistulae. *Ann Vasc Surg* 2014; **28**:1782-8.

[124] Kuo LW, Lin CH. Endovascular salvage for contained rupture of infected thoracic aortic aneurysm with esophageal fistula. *Ann Vasc Surg* 2014; **28**:742.e13-5.

[125] Lee CW, Kao WY, Weng SH, Liu IF. Esophageal submucosal tumor－a red flag in patients receiving thoracic aortic aneurysmal stent-graft. *Korean Circ J* 2014; **44**: 444-5.

[126] Matsuda Y, Yoshimura H, Fukuda Y, et al. Aortic squamous metaplasia in a patient with aortoesophageal fistula secondary to thoracic aortic aneurysm: an autopsy case. *Pathol Int* 2014; **64**: 173-7.

[127] Rawala MS, Ali R, Nanjundappa A. Aortoesophageal fistula after thoracic endovascular aortic stent-graft placement: a potential fatal complication [Abstract]. *Cardiology* 2014; **128**:125.

[128] Seto T, Fukui D, Tanaka H, et al. Tracheo-bronchial obstruction and esophageal perforation after TEVAR for thoracic aortic rupture. *Ann Vasc Dis* 2014; **7**:421-5.

[129] Genuis ED, Kim DJ. A case of secondary aortoesophageal fistula. *J Emerg Med* 2015; **49**: e193-4.

[130] Hackl G, Portugaller RH, Fickert P. Emergency double-stenting and surgery for the successful management of massive upper gastrointestinal bleeding caused by mycotic aortic aneurysm. *Clin Gastroenterol Hepatol* 2015; **13**: e171-2.

[131] He S, Chen X, Zhou X, Hu Q, Ananda S, Zhu S. Sudden death due to traumatic ascending aortic pseudoaneurysms ruptured into the esophagus: 2 case reports. *Medicine* 2015; **94**: e716.

[132] Kawamoto S, Sato M, Motoyoshi N, et al. Outcomes of a staged surgical treatment strategy for aortoesophageal fistula. *Gen Thorac Cardiovasc Surg* 2015; **63**:147-52.

[133] Luehr M, Etz CD, Nozdrzykowski M, et al. Emergency open surgery for aorto-oesophageal and aorto-bronchial fistulae after thoracic endovascular aortic repair: a single-centre experience dagger. *Eur J Cardiothorac Surg* 2015; **47**: 374-82.

[134] Nayak RD, Paul M, Valooran G, Varghese R. Emergent endovascular stent grafting for saccular arch aneurysm complicated by aorto-esophageal fistula. *Indian Heart J* 2015; **67 (Suppl 3)**: S60-3.

[135] Rodriguez A, Lipka S, Shiani A, Gill JA. Successful aortoesophageal fistulous tract closurse using endoscopic clips and endoloop® after previously failed endoscopic therapy [Abstract]. *Am J Gastroenterol* 2015; **110**: S227.

[136]Akin M, Yalcinkaya T, Alkan E, Arslan G, Tuna Y, Yildirim B. A cause of mortal massive upper gastrointestinal bleeding: aortoesophageal fistula. *Med Arch* 2016; **70**: 79-81.

[137] Cheng L, Zhu J, Liu X, et al. A successful three-ztage zurgical treatment for aortoesophageal fistula after thoracic endovascular aortic repair and esophageal stent repair. *Ann Thorac Surg* 2016; **102**: e503-5.

[138] Crisan D, Crisan F, Lazar CS, Pop B. Lessons from autopsies: An unusual cause of massive upper digestive bleeding [Abstract]. *Virchows Archiv* 2016; **469**: S244.

[139] Georvasili VK, Bali C, Peroulis M, et al. Management of an aorto-esophageal fistula, complicating a descending thoracic aortic aneurysm endovascularly repaired. *Gen Thorac Cardiovasc Surg* 2016; **64**: 216-9.

[140] Hakim S, Gjeorgjievski M, Garg L, Orosey M, Desai T. Atypical aortoesophageal fistula with atypical and delayed presentation and negative imaging studies. *Case Rep Gastrointest Med* 2016; **2016**: 7219034.

[141] Končar IB, Dragaš M, Sabljak P, Peško P, Marković M, Davidović L. Aortoesophageal and aortobronchial fistula caused by Candida Albicans after thoracic endovascular aortic repair. *Vojnosanit Pregl* 2016; **73**: 864-7.

[142] Kouritas VK, Dedeilias P, Sotiriou K, Klimopoulos S. Delayed presentation of aortoesophageal fistula after endovascular repair. *Asian Cardiovasc Thorac Ann* 2016; 24: 51-3.

[143] Lee JH, Na B, Hwang Y, Kim YH, Park IK, Kim KH. Surgical management of aorto-esophageal fistula as a late complication after graft replacement for acute aortic dissection. *Korean J Thorac Cardiovasc Surg* 2016; **49**: 54-8.

[144] Martínez-González J, Cañete-Ruiz A, Aicart-Ramos M. Dysphagia and hematemesis: a rare presentation of a vascular complication. *Gastroenterol Hepatol* 2016; 39: 24-5.

[145] Nazarewicz GV, Jain R. Upper gastrointestinal bleeding caused by aortoesophageal fistula. *Clin Gastroenterol Hepatol* 2016; **14**: A22.

[146] Okwara CJ, Petrasek J, Gibson M, Burstein E. Secondary aortoesophageal fistula associated with aneurysmal graft infection by Coxiella burnetii. *ACG Case Rep J* 2016; **3**: 169-71.

[147] Omura A, Yoshida M, Koda Y, Mukohara N. Surgical management without resection of the oesophagus for aorto-oesophageal fistula secondary to aortic arch aneurysm rupture. *Interact Cardiovasc Thorac Surg* 2016; **23**: 985-7.

[148] Tanaka A, Sakamoto T, Matsumori M, et al. A cure with successful staged treatment of aortoesophageal fistula. *Gen Thorac Cardiovasc Surg* 2016; **64**: 28-30.

[149] Tao M, Shlomovitz E, Darling G, Roche-Nagle G. Secondary aorto-esophageal fistula after thoracic aortic aneurysm endovascular repair treated by covered esophageal stenting. *World J Clin Cases* 2016; **4**: 233-7.

[150] Aday U, Çetin DA, Çiyiltepe H, Gündeş E, Bozdağ E, Senger AS. Cause of mortality in aortoesophageal fistula: oesophageal sepsis. A case report. *Prz Gastroenterol* 2017; **12**: 222-5.

[151] Chan D, O'Donnell C, Parsons S. Sudden cardiac death from aortoesophageal fistula: an autopsy case report of a rare complication of thoracic aortic aneurysm. *Forensic Sci Med Pathol* 2017; **13**: 504-7.

[152] Da Silva E WGM, Caiafa JS, Gress MHT, et al. Aortoesophageal fistula: case report. *Vascular* 2017; **25**: 43-4.

[153] Fernandez R, Malaisrie SC, Hoel A, Bharat A. Aortoesophageal fistula resulting from aortic endograft migration. *J Thorac Cardiovasc Surg* 2017; **154**: 785-6. e1.

[154] Nakamura T, Yamamoto M, Yamazato T, et al. Surgical strategy of esophageal resection and reconstruction for aortoesophageal fistula. *Dis Esophagus* 2017; **30**: 1-7.

[155] Nozdrzykowski M, Garbade J, Leinung S, Schmidt A, Mohr FW, Borger MA. Thoracic endovascular aortic repair for aortoesophageal fistula after covered rupture of aortic homograft. *Aorta* 2017; **5**: 96-100.

[156] Qaja E, Sivakumar M, Saleemi M. An unusual case of massive hematemesis caused by aorto-esophageal fistula due to mycotic aneurysm of mid-thoracic aorta in a patient without prior aortic instrumentation. *J Surg Case Rep* 2017; **2017**: rjx084.

[157] Schierz IAM, Piro E, Giuffre M, Pinello G, Corsello G. Dilated azygos arch mimicking an aortic arch anomaly during thoracic surgery. *Early Hum Dev* 2017; **111**: 20-2.

[158] Sladojevic M, Bjelovic M, Ilic N, et al. Open surgical treatment of secondary aortoesophageal and aortobronchial fistula after thoracic endovascular aortic repair and esophagocoloplasty in a second procedure. *Ann Vasc Surg* 2017; **44**: 417.e11-6.

[159] Spitaels R, Jacob W, Janssens F, et al. Thoracic aortic aneurysm complicated by secondary aortoesophageal fistula after thoracic endovascular aortic repair : a case report. *Acta Gastroenterol Belg* 2017; **80**: 525-7.

[160] Yamamoto M, Nishimori H, Iguchi M, Orihashi K. Pathological investigation of graft-related oesophageal fistula. *Interact Cardiovasc Thorac Surg* 2017; **24**: 813-4.

[161] Yamanaka K, Shiiya N, Washiyama N, Sato M. Secondary graft-oesophageal fistula after total arch replacement. *Interact Cardiovasc Thorac Surg* 2017; **25**: 331-2.

[162] Dreher GJ, Kronfeld PHO, Paniz LGP, et al. Endovascular management of aortoesophageal fistulae with intercostal muscle flap interposition: case report [Abstract]. *Vasc Endovasc Surg* 2018; **52**: S40-1.

[163] Idhrees AM, Jacob A, Velayudhan BV. An aorto-oesophageal fistula following endograft: sealing of fistulae with omentum and replacement of the aorta. *Interact Cardiovasc Thorac Surg* 2018; **26**: 516-8.

[164] Karkos CD, Goulis I. An unusual cause of haematemesis. *Eur J Vasc Endovasc Surg* 2018; **55**: 656.

[165] Majumder A, Dharmaraj RB. Successful endovascular management of a case of aorto-oesophageal fistula presenting as life threatening upper gastrointestinal bleed. *EJVES Short Rep* 2018; **39**: 29-32.

[166] Matsumiya K, Nishihara S, Miyagami H. Endoscopic injection of alpha-cyanoacrylate monomer (A-CA) for a thoracic endovascular repair (TEVAR) with a stent graft for an aortoesophageal fistula [Abstract]. *J Am Coll Cardiol* 2018; **71**: A2164.

[167] Rawala MS, Badami V, Rizvi SB, Nanjundappa A. Aortoesophageal fistula: a fatal complication of thoracic endovascular aortic stent-graft placement. *Am J Case Rep* 2018; **19**: 1258-61.

[168] Sharma M, Singh P, Kirnake V, Toshniwal J, Chopra A. Dysphagia aortica: Emerging role of endoscopic ultrasound (with videos). *Endosc Ultrasound* 2018; **7**: 343-6.

[169] So K, Smith CR, Faroqui NM, et al. Control of aortoesophageal fistula using endoscopic and endovascular techniques: a palliative intervention. *Am Surg* 2018; **84**: e47-9.

[170] Takei N, Kunieda T, Kumada Y, Murayama M. Perigraft abscess subsequent to aortoesophageal fistula. *Intern Med* 2018; **57**: 3255-9.

[171] Usai MV, Gottschalk A, Schönefeld T, Schaefers JF, Torsello GB, Rukosujew A. Late-onset aortoesophageal fistula after treatment of a chronic type B aortic dissection with a three-step approach. *J Vasc Surg Cases Innov Tech* 2018; **4**: 50-3.

[172] Yang G, Wang X, Tang J. Stomach for esophageal replacement after two-stage surgeries of aortoesophageal fistula. *Int J Clin Exp Med* 2018; **11**: 11342-5.

[173] Yang Y, Hu D, Peng D. Primary aortoesophageal fistula: a fatal outcome. *Am J Emerg Med* 2018; **36**: 343.e1-3.

[174] Amandine D, Dominique V, Thomas D, Pascal D, Nicolas M. A giant thoracic aortic aneurysm causing multiple fistulas and mediastinitis. *Anaesth Crit Care Pain Med* 2019; **38**:279-80.

[175] Gupta A, Poptani A, Satwik A. Primary aortoesophageal fistula: A rare cause of upper gastrointestinal bleeding. *Indian J Gastroenterol* 2019; **38**: 83-4.

[176] Hwang SH, Cho JW, Bae CH, Jang JS. Staged surgical treatment of primary aortoesophageal fistula. *Korean J Thorac Cardiovasc Surg* 2019; **52**: 182-5.

[177] Kamigaichi A, Hamai Y, Emi M, et al. Three-step surgical treatment of aortoesophageal fistula after thoracic endovascular aortic repair: A case report. [*Int J Surg Case Rep*](https://www-ncbi-nlm-nih-gov.manchester.idm.oclc.org/pubmed/?term=Three-step+surgical+treatment+of+aortoesophageal+fistula+after+thoracic+endovascular+aortic+repair%3A+A+case+report) 2019; **65**: 221-4.

[178] Karb DB, Mansoor E, Sullivan J, Gollamudi J, Wong RCK. Atypical presentation of aortoesophageal fistula without hemorrhage. *ACG Case Rep J* 2019; **6**:e00004.

[179] Kayashima A, Mori H, Okuzawa A, et al. An esophageal ulcer associated with a thoracoabdominal aortic aneurysm. *Case Rep Gastroenterol* 2019; **13**:214-8.

[180] Mori S, Kimura S, Ro A, et al. Two autopsy cases of rupture of the aorta by fistula formation after thoracic endovascular aortic repair and open stent-grafting on aortic arch aneurysm. *Cardiovasc Pathol* 2019; **39**: 61-6.

[181] Slieker FJB, Ruurda JP, Hazenberg CEVB. Esophageal resection, tastric tube reconstruction, and omental flap coverage of iatrogenic aortoesophageal fistula after secondary thoracic stentgraft infection: a case report. [*Ann Vasc Surg*](https://www-ncbi-nlm-nih-gov.manchester.idm.oclc.org/pubmed/?term=Esophageal+resection%2C+tastric+tube+reconstruction%2C+and+omental+flap+coverage+of+iatrogenic+aortoesophageal+fistula+after+secondary+thoracic+stentgraft+infection%3A+a+case+report) 2019; **59**: 314.e311-4.

[182] Stanger E, Johnson AM, Juneja Mucci J. Novel management of an aortoesophageal fistula prior to definitive surgical repair. *Clin Med Insights Case Rep* 2019; **12**: 1179547619839710.

[183] Wu Z, Ma Y. Man with tarry stool. *Am J Med Sci* 2019; **358**: e7.

[184] Ito T, Nobuoka T, Sato H, et al. Total surgical repair for secondary aortoesophageal fistula: two case reports. *Gen Thorac Cardiovasc Surg* 2020; **68**: 290-4.
